# The complete chloroplast genome sequence of *Pinus kesiya var. langbianensis*

**DOI:** 10.1080/23802359.2019.1627929

**Published:** 2019-07-11

**Authors:** Yi Wang, Xiaolong Yuan, Wei Chen, Jiang Li

**Affiliations:** Laboratory of Forest Plant Cultivation and Utilization, Yunnan Academy of Forestry, Kunming, People’s Republic of China

**Keywords:** *Pinus kesiya var. langbianensis*, chloroplast, Illumina sequencing, phylogenetic analysis

## Abstract

*Pinus kesiya var. langbianensis* is an important oleoresin resource tree species in Yunnan province, China. In this study, the complete chloroplast genome (cpDNA) sequence of *P. kesiya var. langbianensis* was determined from Illumina HiSeq pair-end sequencing data. The cpDNA is 119,780 bp in length, contains a large single copy region (LSC) of 65,863 bp and a small single copy region (SSC) of 53,117 bp, which were separated by a pair of inverted repeat (IR) regions of 400 bp. The genome contains 113 genes, including 73 protein-coding genes, 4 ribosomal RNA genes, and 36 transfer RNA genes. The overall GC content of the whole genome is 38.5%, and the corresponding values of the LSC, SSC, and IR regions are 37.9, 39.4, and 35.5%, respectively. Further phylogenomic analysis showed that *P. kesiya var. langbianensis* clustered together with *Pinus kesiya, Pinus densata*, and *Pinus taiwanensis*.

*Pinus kesiya var. langbianensis* is a coastal species in Asia, and it is mainly distributed in southwest China and southeast Asia (Zhao et al. 2018). It is an important oleoresin resource tree species in Yunnan province, China (Yin et al. 2005). The output of pine oleoresin of *P. kesiya var. langbianensis* is 179,100,000 kg every year (Wang et al. 2018). The forestland area of Simao pine is 0.56 million hm^2^ (Li et al. 2017). However, there has been no genomic studies on *P. kesiya var. langbianensis*.

Herein, we reported and characterized the complete *P. kesiya var. langbianensis* plastid genome (MK782762). One *P. kesiya var. langbianensis* individual (specimen number: 2016050134) was collected from Jinggu, Puer, Yunnan Province of China (23°47′47.8″ N, 100°27′55.8″ E). The specimen is stored at Yunnan Academy of Forestry Herbarium. DNA was extracted from its fresh pine needle using DNeasy Plant Kit (Qiagen, Hilden, Germany).

Paired-end reads were sequenced by using Illumina HiSeq system (Illumina, San Diego, CA). In total, about 21.87 million high-quality clean reads were generated with adaptors trimmed. Aligning, assembly, and annotation were conducted by CLC de novo assembler (CLC Bio, Aarhus, Denmark), BLAST, GeSeq (Tillich et al. 2017), and Geneious v11.0.5 (Biomatters Ltd, Auckland, New Zealand). To confirm the phylogenetic position of *P. kesiya var. langbianensis*, other 16 species of genus *Pinus* from NCBI were aligned using MAFFT v.7 (Katoh and Standley 2013) and maximum-likelihood (ML) bootstrap analysis was conducted using RAxML (Stamatakis 2006); bootstrap probability values were calculated from 1000 replicates. *Picea asperata* (KY204451) was served as the out-group.

The complete *P. kesiya var. langbianensis* plastid genome is a circular DNA molecule with a length of 119,780 bp, and contains a large single copy region (LSC) of 65,863 bp and a small single copy region (SSC) of 53,117 bp, which were separated by a pair of inverted repeat (IR) regions of 400 bp. The overall GC content of the whole genome is 38.5%, and the corresponding values of the LSC, SSC, and IR regions are 37.9, 39.4, and 35.5%, respectively. The plastid genome contained 113 genes, including 73 protein-coding genes, 4 ribosomal RNA genes, and 36 transfer RNA genes. Phylogenetic analysis showed that *P. kesiya var. langbianensis* clustered together with *Pinus kesiya*, *Pinus densata*, and *Pinus taiwanensis* (Figure 1), which indicated the phylogenesis classification of *P. kesiya var. langbianensis*. The determination of the complete plastid genome sequences provided new molecular data to illuminate the *Pinus* evolution.

**Figure 1.**
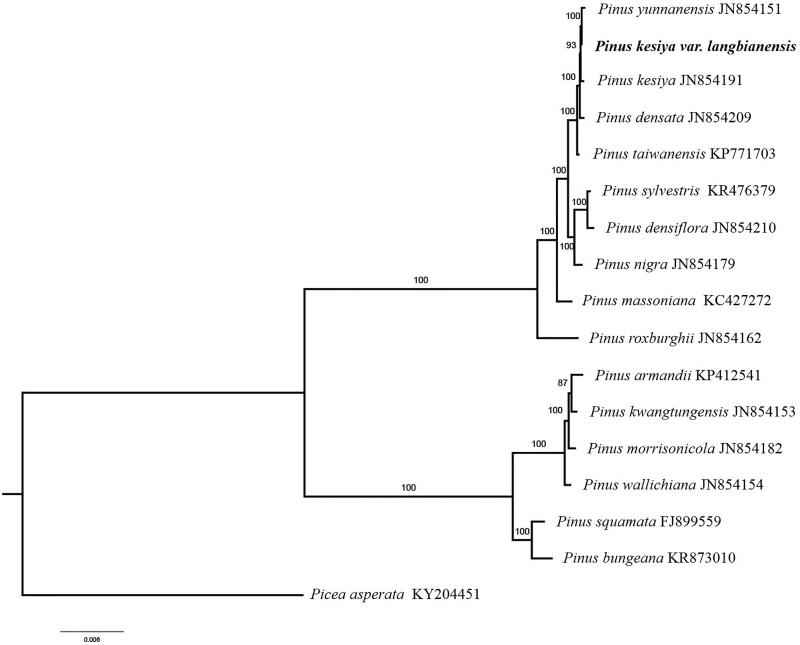
The maximum-likelihood tree based on the 16 chloroplast genomes of genus *Pinus*. The bootstrap value based on 1000 replicates is shown on each node.
